# Crystal structure of 4-bromo-2-(1*H*-imidazo[4,5-*b*]pyridin-2-yl)phenol

**DOI:** 10.1107/S2056989015022197

**Published:** 2015-11-28

**Authors:** Kamel Ouari

**Affiliations:** a Laboratoire d’Electrochimie, d’Ingénierie Moléculaire et de Catalyse Redox, Faculty of Technology, University of Ferhat Abbas Sétif-1, 19000 Sétif, Algeria

**Keywords:** crystal structure, 2,3-di­amino­pyridine, 5-bromo-2-hy­droxy-1-salycilaldehyde, hydrogen bonding

## Abstract

In the title compound, C_12_H_8_BrN_3_O, the 4-bromo­phenol ring is coplanar with the planar imidazo[4,5-*b*]pyridine moiety (r.m.s deviation = 0.015 Å), making a dihedral angle of 1.8 (2)°. There is an intra­molecular O—H⋯N hydrogen bond forming an *S*(6) ring motif. In the crystal, mol­ecules are linked *via* N—H⋯N and O—H⋯Br hydrogen bonds, forming undulating sheets parallel to (10-2). The sheets are linked by π–π inter­actions [inter-centroid distance = 3.7680 (17) Å], involving inversion-related mol­ecules, forming a three-dimensional structure.

## Related literature   

For some recent examples of transition metal complexes of Schiff bases, see: Ouari *et al.* (2015*b* ▸); Benghanem *et al.* (2012 ▸); Basu *et al.* (2010 ▸). For the biological activity of Schiff bases, see: Yıldız *et al.* (2015 ▸); Salama *et al.* (2015 ▸); Zayed *et al.* (2015 ▸). For the photoluminescence of the title compound, see: Köse *et al.* (2015 ▸); Pal *et al.* (2015 ▸); Ray *et al.* (2014 ▸). For the literature method used to prepare the title compound, see: Ouari *et al.* (2015*a* ▸). For the crystal structure of a related compound, see: Belguedj *et al.* (2015 ▸).
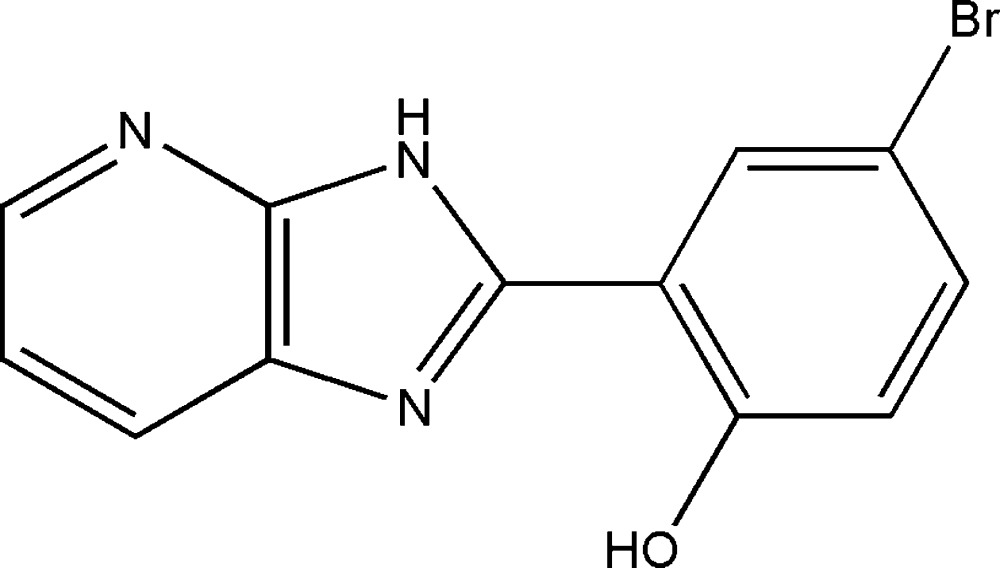


## Experimental   

### Crystal data  

C_12_H_8_BrN_3_O
*M* *_r_* = 290.12
Monoclinic, 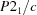

*a* = 5.5906 (3) Å
*b* = 12.9032 (7) Å
*c* = 14.7622 (6) Å
β = 102.836 (3)°
*V* = 1038.28 (9) Å^3^
*Z* = 4
Mo *K*α radiation
μ = 3.94 mm^−1^
*T* = 193 K
0.25 × 0.20 × 0.05 mm

### Data collection  

Nonius KappaCCD diffractometer
Absorption correction: multi-scan (*MULABS* in *PLATON*; Spek, 2009 ▸) *T*_min_ = 0.457, *T*_max_ = 0.721
8584 measured reflections
3017 independent reflections
1977 reflections with *I* > 2σ(*I*)
*R*_int_ = 0.066

### Refinement  

*R*[*F* ^2^ > 2σ(*F* ^2^)] = 0.043
*wR*(*F* ^2^) = 0.111
*S* = 1.02
3017 reflections
159 parameters
H atoms treated by a mixture of independent and constrained refinement
Δρ_max_ = 0.52 e Å^−3^
Δρ_min_ = −0.84 e Å^−3^

### 

Data collection: *COLLECT* (Nonius, 1998 ▸); cell refinement: *DENZO* (Nonius, 1998 ▸); data reduction: *DENZO*; program(s) used to solve structure: *SHELXS97* (Sheldrick, 2008 ▸); program(s) used to refine structure: *SHELXL97* (Sheldrick, 2008 ▸); molecular graphics: *Mercury* (Macrae *et al.*, 2008 ▸); software used to prepare material for publication: *SHELXL97* and *PLATON* (Spek, 2009 ▸).

## Supplementary Material

Crystal structure: contains datablock(s) I, Global. DOI: 10.1107/S2056989015022197/su5238sup1.cif

Structure factors: contains datablock(s) I. DOI: 10.1107/S2056989015022197/su5238Isup2.hkl

Supporting information file. DOI: 10.1107/S2056989015022197/su5238Isup3.cml

**Fig. 1.**
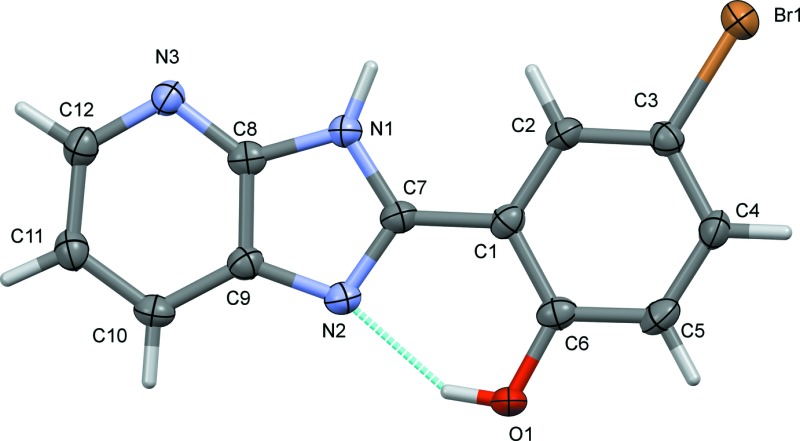
The molecular structure of the title compound, with atom labelling scheme. Displacement ellipsoids are drawn at the 50% probability level. The intramolecular O-H···N hydrogen bond is shown as a dashed line (see Table 1).

**Fig. 2.**
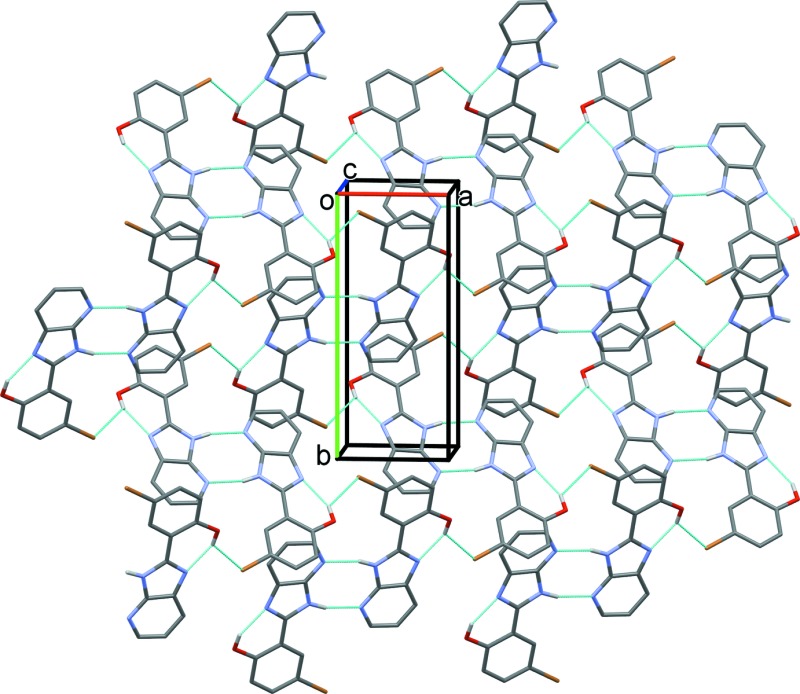
A view along the c axis of the crystal packing of the title compound. The hydrogen bonds are shown as dashed lines (see Table 1), and H atoms not involved in these interactions have been omitted for clarity.

. DOI: 10.1107/S2056989015022197/su5238fig1.tif
The mol­ecular structure of the title compound, with atom labelling scheme. Displacement ellipsoids are drawn at the 50% probability level. The intra­molecular O-H⋯N hydrogen bond is shown as a dashed line (see Table 1).

c . DOI: 10.1107/S2056989015022197/su5238fig2.tif
A view along the *c* axis of the crystal packing of the title compound. The hydrogen bonds are shown as dashed lines (see Table 1), and H atoms not involved in these inter­actions have been omitted for clarity.

CCDC reference: 1437912

Additional supporting information:  crystallographic information; 3D view; checkCIF report

## Figures and Tables

**Table 1:** Hydrogen-bond geometry (Å, °)

*D*—H⋯*A*	*D*—H	H⋯*A*	*D*⋯*A*	*D*—H⋯*A*
O1—H1⋯N2	0.84	1.90	2.640 (3)	147
O1—H1⋯Br1^i^	0.84	2.91	3.478 (2)	127
N1—H1*N*⋯N3^ii^	0.92 (4)	2.11 (4)	3.010 (4)	168 (3)

Symmetry codes: (i) 
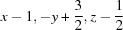
; (ii) 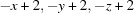.

## supplementary crystallographic information

### S1. Comment

Coordination chemistry of transition metal complexes with Schiff base ligands is an important and fascinating branch of chemistry (Ouari *et al.*, 2015b; Benghanem *et al.*, 2012; Basu *et al.*, 2010). A literature survey revealed that this kind of compound possesses diverse biological activities such as anti­biotic (Yıldız *et al.*, 2015) and anti­microbial (Salama *et al.*, 2015; Zayed *et al.*, 2015). The photoluminescence of the title compound has been reported (Köse *et al.*, 2015; Pal *et al.*, 2015; Ray *et al.*, 2014).

The molecular structure of the title compound is shown in Fig. 1. The bond distances and angles are normal and similar to those in related compounds (Belguedj *et al.*, 2015).

In the crystal, molecules are linked via N—H···N and O—H···Br hydrogen bonds forming undulating sheets parallel to (102); see Table 1 and Fig. 2. The sheets are linked by π-π inter­actions [Cg2···Cg3^i^ = 3.7680 (17) Å, Cg2 and Cg3 are the centroids of rings N3/C8—C12 and C1—C6, symmetry code: (i) - x + 1, - y + 2, - z + 2], forming a three-dimensional structure.

### S2. Synthesis and crystallisation

The title compound was prepared following a literature method (Ouari *et al.*, 2015a). To a MeOH solution (15 ml) of 5-bromo­salicyl­aldehyde (0.122 g, 1 mmol) was added drop wise to a MeOH solution (5 ml) of 2,3-di­amino­pyridine (0.109 g, 1 mmol). The mixture was refluxed with constant stirring under a nitro­gen atmosphere for 3 h, yielding an abundant orange precipitate that was collected by filtration. The product was washed with methanol (3 × 5 ml) then with di­ethyl ether (3 × 5 ml) and dried under vacuum for 4 h. Orange crystals of the title compound, suitable for X-ray diffraction analysis, were obtained after two weeks by slow evaporation of the DMSO solvent (yield: 70%; m.p.: 528-531 K).

### S3. Refinement

Crystal data, data collection and structure refinement details are summarized in Table 2. The iminium H atom was located from a difference Fourier map and freely refined. The OH and C-bound H atoms were included in calculated positions and treated as riding atoms: O—H = 0.82 Å, C—H = 0.95-0.99 Å with U_iso_(H) = 1.5U_eq_(O) and 1.2U_eq_(C).

### Crystal data

**Table:**

C_12_H_8_BrN_3_O	*F*(000) = 576
*M**_r_* = 290.12	*D*_x_ = 1.856 Mg m^−^^3^
Monoclinic, *P*2_1_/*c*	Mo *K*α radiation, λ = 0.71073 Å
Hall symbol: -P 2ybc	Cell parameters from 4475 reflections
*a* = 5.5906 (3) Å	θ = 1.0–30.0°
*b* = 12.9032 (7) Å	µ = 3.94 mm^−^^1^
*c* = 14.7622 (6) Å	*T* = 193 K
β = 102.836 (3)°	Plate, orange
*V* = 1038.28 (9) Å^3^	0.25 × 0.20 × 0.05 mm
*Z* = 4	

### Data collection

**Table:**

Nonius KappaCCD diffractometer	3017 independent reflections
Radiation source: sealed tube	1977 reflections with *I* > 2σ(*I*)
Graphite monochromator	*R*_int_ = 0.066
phi and ω scans	θ_max_ = 30.0°, θ_min_ = 2.1°
Absorption correction: multi-scan (*MULABS* in *PLATON*; Spek, 2009)	*h* = −7→4
*T*_min_ = 0.457, *T*_max_ = 0.721	*k* = −17→18
8584 measured reflections	*l* = −20→19

### Refinement

**Table:**

Refinement on *F*^2^	Primary atom site location: structure-invariant direct methods
Least-squares matrix: full	Secondary atom site location: difference Fourier map
*R*[*F*^2^ > 2σ(*F*^2^)] = 0.043	Hydrogen site location: inferred from neighbouring sites
*wR*(*F*^2^) = 0.111	H atoms treated by a mixture of independent and constrained refinement
*S* = 1.02	*w* = 1/[σ^2^(*F*_o_^2^) + (0.045*P*)^2^ + 0.5334*P*] where *P* = (*F*_o_^2^ + 2*F*_c_^2^)/3
3017 reflections	(Δ/σ)_max_ = 0.002
159 parameters	Δρ_max_ = 0.52 e Å^−^^3^
0 restraints	Δρ_min_ = −0.84 e Å^−^^3^

### Special details

**Table:**

Geometry. All s.u.'s (except the s.u. in the dihedral angle between two l.s. planes) are estimated using the full covariance matrix. The cell s.u.'s are taken into account individually in the estimation of s.u.'s in distances, angles and torsion angles; correlations between s.u.'s in cell parameters are only used when they are defined by crystal symmetry. An approximate (isotropic) treatment of cell s.u.'s is used for estimating s.u.'s involving l.s. planes.
Refinement. Refinement of *F*^2^ against ALL reflections. The weighted *R*-factor *wR* and goodness of fit *S* are based on *F*^2^, conventional *R*-factors *R* are based on *F*, with *F* set to zero for negative *F*^2^. The threshold expression of *F*^2^ > 2σ(*F*^2^) is used only for calculating *R*-factors(gt) *etc*. and is not relevant to the choice of reflections for refinement. *R*-factors based on *F*^2^ are statistically about twice as large as those based on *F*, and *R*- factors based on ALL data will be even larger.

### Fractional atomic coordinates and isotropic or equivalent isotropic displacement parameters (Å^2^)

**Table:**

	*x*	*y*	*z*	*U*_iso_*/*U*_eq_	
Br1	0.83482 (7)	0.58489 (3)	1.18198 (2)	0.03255 (13)	
O1	0.0419 (4)	0.75774 (17)	0.87477 (15)	0.0274 (5)	
H1	0.0891	0.8104	0.8502	0.041*	
N1	0.7079 (5)	0.9288 (2)	0.95213 (18)	0.0226 (6)	
N2	0.3311 (5)	0.91524 (19)	0.85876 (18)	0.0249 (6)	
N3	0.8399 (5)	1.09033 (19)	0.89394 (18)	0.0242 (6)	
C1	0.4545 (6)	0.7733 (2)	0.9701 (2)	0.0231 (7)	
C2	0.6365 (6)	0.7295 (2)	1.0408 (2)	0.0248 (7)	
H2	0.7917	0.7625	1.0592	0.030*	
C3	0.5911 (6)	0.6389 (2)	1.0835 (2)	0.0245 (7)	
C4	0.3660 (6)	0.5889 (2)	1.0569 (2)	0.0276 (7)	
H4	0.3362	0.5266	1.0868	0.033*	
C5	0.1866 (6)	0.6303 (3)	0.9869 (2)	0.0299 (8)	
H5	0.0333	0.5959	0.9684	0.036*	
C6	0.2276 (6)	0.7220 (2)	0.9429 (2)	0.0247 (7)	
C7	0.4972 (6)	0.8720 (2)	0.9272 (2)	0.0229 (7)	
C8	0.6765 (6)	1.0143 (2)	0.8952 (2)	0.0222 (6)	
C9	0.4404 (6)	1.0061 (2)	0.8373 (2)	0.0233 (7)	
C10	0.3592 (6)	1.0841 (2)	0.7730 (2)	0.0265 (7)	
H10	0.1997	1.0834	0.7336	0.032*	
C11	0.5249 (7)	1.1633 (2)	0.7697 (2)	0.0287 (7)	
H11	0.4796	1.2182	0.7264	0.034*	
C12	0.7572 (6)	1.1635 (3)	0.8290 (2)	0.0281 (7)	
H12	0.8647	1.2189	0.8233	0.034*	
H1N	0.843 (8)	0.912 (3)	0.998 (3)	0.034 (10)*	

### Atomic displacement parameters (Å^2^)

**Table:**

	*U*^11^	*U*^22^	*U*^33^	*U*^12^	*U*^13^	*U*^23^
Br1	0.0314 (2)	0.0314 (2)	0.0320 (2)	−0.00049 (16)	0.00116 (14)	0.00779 (15)
O1	0.0181 (12)	0.0281 (12)	0.0317 (12)	−0.0034 (9)	−0.0036 (9)	0.0059 (10)
N1	0.0197 (14)	0.0238 (14)	0.0231 (13)	−0.0013 (11)	0.0025 (11)	0.0028 (11)
N2	0.0211 (14)	0.0251 (14)	0.0264 (13)	−0.0024 (12)	0.0010 (11)	−0.0003 (11)
N3	0.0236 (14)	0.0224 (14)	0.0270 (14)	−0.0024 (11)	0.0063 (11)	0.0007 (11)
C1	0.0212 (17)	0.0236 (16)	0.0258 (16)	−0.0015 (12)	0.0079 (13)	−0.0015 (13)
C2	0.0195 (17)	0.0261 (17)	0.0281 (16)	−0.0050 (13)	0.0038 (13)	−0.0016 (13)
C3	0.0252 (18)	0.0223 (16)	0.0253 (16)	0.0027 (13)	0.0037 (13)	0.0003 (13)
C4	0.0292 (18)	0.0198 (15)	0.0343 (18)	−0.0048 (14)	0.0079 (14)	0.0019 (14)
C5	0.0261 (19)	0.0254 (18)	0.0381 (19)	−0.0082 (14)	0.0070 (15)	−0.0064 (15)
C6	0.0212 (17)	0.0274 (17)	0.0257 (16)	−0.0006 (13)	0.0055 (13)	−0.0049 (13)
C7	0.0192 (16)	0.0259 (16)	0.0235 (15)	−0.0018 (13)	0.0044 (13)	−0.0017 (13)
C8	0.0212 (16)	0.0251 (16)	0.0211 (15)	0.0012 (13)	0.0067 (12)	−0.0042 (13)
C9	0.0220 (17)	0.0248 (16)	0.0235 (15)	−0.0014 (13)	0.0056 (13)	−0.0007 (13)
C10	0.0232 (17)	0.0309 (17)	0.0232 (15)	0.0006 (15)	0.0004 (12)	−0.0014 (14)
C11	0.035 (2)	0.0248 (17)	0.0261 (17)	0.0003 (15)	0.0066 (14)	0.0036 (14)
C12	0.0287 (18)	0.0255 (17)	0.0315 (18)	−0.0025 (14)	0.0100 (14)	0.0014 (14)

### Geometric parameters (Å, º)

**Table:**

Br1—C3	1.891 (3)	C2—H2	0.9500
O1—C6	1.356 (4)	C3—C4	1.391 (5)
O1—H1	0.8400	C4—C5	1.378 (5)
N1—C7	1.366 (4)	C4—H4	0.9500
N1—C8	1.375 (4)	C5—C6	1.393 (5)
N1—H1N	0.92 (4)	C5—H5	0.9500
N2—C7	1.333 (4)	C8—C9	1.407 (4)
N2—C9	1.390 (4)	C9—C10	1.390 (4)
N3—C8	1.344 (4)	C10—C11	1.387 (5)
N3—C12	1.352 (4)	C10—H10	0.9500
C1—C2	1.405 (4)	C11—C12	1.395 (5)
C1—C6	1.408 (4)	C11—H11	0.9500
C1—C7	1.465 (4)	C12—H12	0.9500
C2—C3	1.378 (4)		
			
C6—O1—H1	109.5	O1—C6—C5	117.2 (3)
C7—N1—C8	106.2 (3)	O1—C6—C1	123.0 (3)
C7—N1—H1N	126 (2)	C5—C6—C1	119.9 (3)
C8—N1—H1N	127 (2)	N2—C7—N1	113.2 (3)
C7—N2—C9	104.9 (3)	N2—C7—C1	122.6 (3)
C8—N3—C12	113.1 (3)	N1—C7—C1	124.3 (3)
C2—C1—C6	118.7 (3)	N3—C8—N1	126.8 (3)
C2—C1—C7	120.7 (3)	N3—C8—C9	126.6 (3)
C6—C1—C7	120.6 (3)	N1—C8—C9	106.6 (3)
C3—C2—C1	120.3 (3)	C10—C9—N2	132.3 (3)
C3—C2—H2	119.8	C10—C9—C8	118.7 (3)
C1—C2—H2	119.8	N2—C9—C8	109.0 (3)
C2—C3—C4	120.8 (3)	C11—C10—C9	116.0 (3)
C2—C3—Br1	119.4 (2)	C11—C10—H10	122.0
C4—C3—Br1	119.9 (2)	C9—C10—H10	122.0
C5—C4—C3	119.6 (3)	C10—C11—C12	121.0 (3)
C5—C4—H4	120.2	C10—C11—H11	119.5
C3—C4—H4	120.2	C12—C11—H11	119.5
C4—C5—C6	120.7 (3)	N3—C12—C11	124.6 (3)
C4—C5—H5	119.6	N3—C12—H12	117.7
C6—C5—H5	119.6	C11—C12—H12	117.7
			
C6—C1—C2—C3	−1.1 (5)	C6—C1—C7—N2	−3.0 (5)
C7—C1—C2—C3	177.2 (3)	C2—C1—C7—N1	−1.0 (5)
C1—C2—C3—C4	0.6 (5)	C6—C1—C7—N1	177.3 (3)
C1—C2—C3—Br1	−177.4 (2)	C12—N3—C8—N1	179.0 (3)
C2—C3—C4—C5	0.1 (5)	C12—N3—C8—C9	0.0 (5)
Br1—C3—C4—C5	178.1 (3)	C7—N1—C8—N3	−178.5 (3)
C3—C4—C5—C6	−0.4 (5)	C7—N1—C8—C9	0.7 (3)
C4—C5—C6—O1	−179.9 (3)	C7—N2—C9—C10	−179.5 (3)
C4—C5—C6—C1	−0.1 (5)	C7—N2—C9—C8	0.3 (4)
C2—C1—C6—O1	−179.4 (3)	N3—C8—C9—C10	−1.6 (5)
C7—C1—C6—O1	2.3 (5)	N1—C8—C9—C10	179.2 (3)
C2—C1—C6—C5	0.8 (5)	N3—C8—C9—N2	178.6 (3)
C7—C1—C6—C5	−177.5 (3)	N1—C8—C9—N2	−0.6 (4)
C9—N2—C7—N1	0.2 (4)	N2—C9—C10—C11	−178.4 (3)
C9—N2—C7—C1	−179.6 (3)	C8—C9—C10—C11	1.9 (4)
C8—N1—C7—N2	−0.6 (4)	C9—C10—C11—C12	−0.8 (5)
C8—N1—C7—C1	179.2 (3)	C8—N3—C12—C11	1.3 (5)
C2—C1—C7—N2	178.7 (3)	C10—C11—C12—N3	−0.9 (5)

### Hydrogen-bond geometry (Å, º)

**Table:**

*D*—H···*A*	*D*—H	H···*A*	*D*···*A*	*D*—H···*A*
O1—H1···N2	0.84	1.90	2.640 (3)	147
O1—H1···Br1^i^	0.84	2.91	3.478 (2)	127
N1—H1*N*···N3^ii^	0.92 (4)	2.11 (4)	3.010 (4)	168 (3)

Symmetry codes: (i) *x*−1, −*y*+3/2, *z*−1/2; (ii) −*x*+2, −*y*+2, −*z*+2.
