# Supplementary figures and images for: Crystal structure of 4-bromo-2-(1H-imidazo[4,5-b]pyridin-2-yl)phenol
Source: Acta Crystallogr E Crystallogr Commun. 2015 Nov 28;71(Pt 12):o991–2. doi: 10.1107/S2056989015022197 (PMC4719939; doi:10.1107/S2056989015022197)

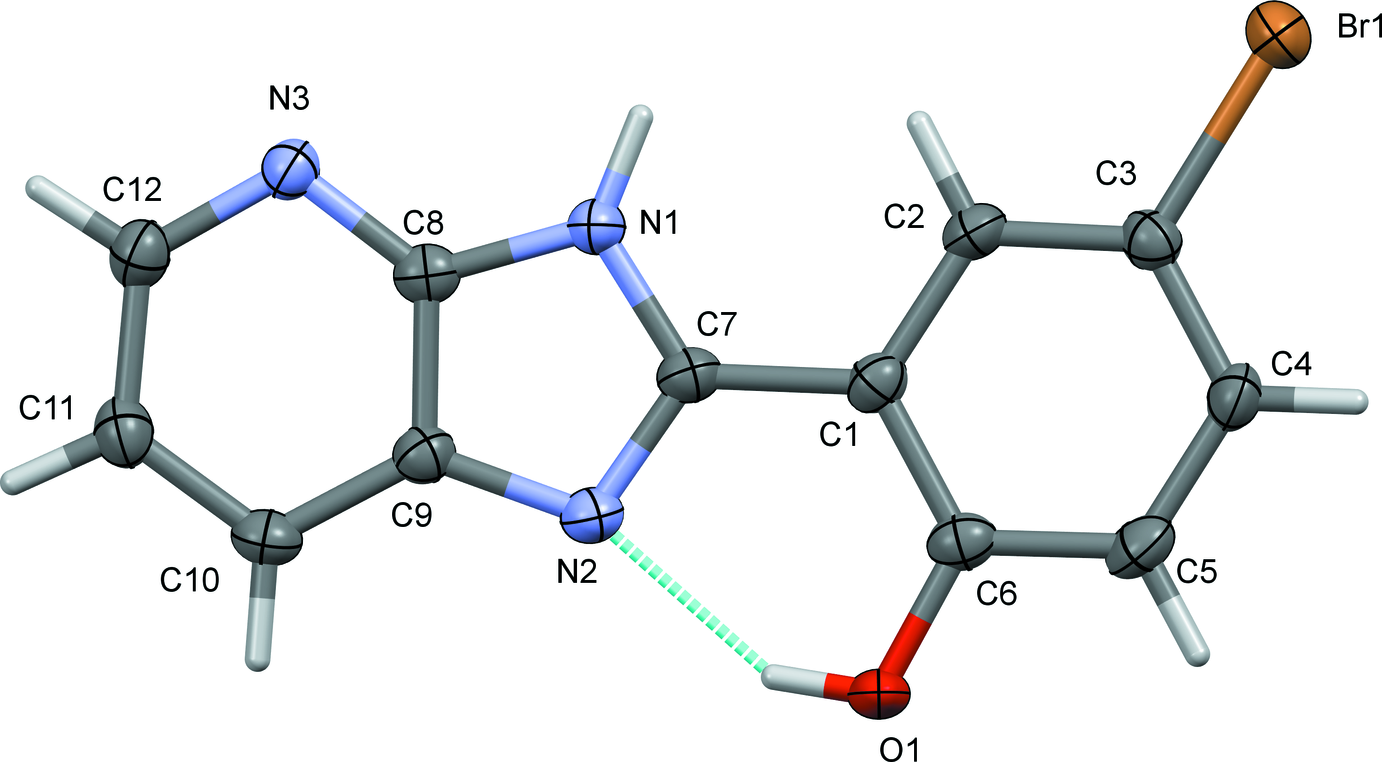

Supplement: Supplementary file 4 [file e-71-0o991-fig1.tif]

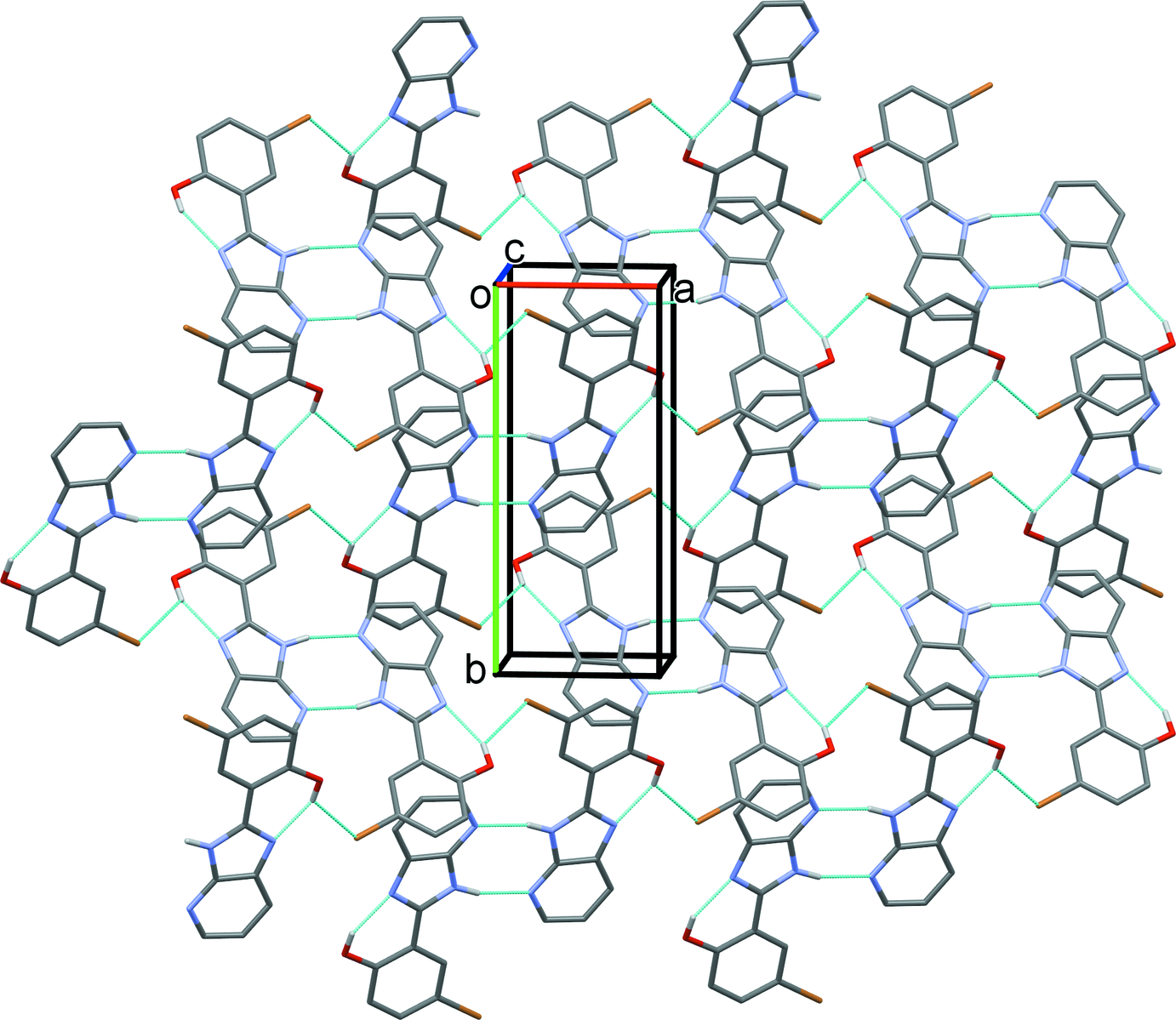

Supplement: Supplementary file 5 [file e-71-0o991-fig2.tif]
